# Supplementary material for: Rare Chromone Derivatives from the Marine-Derived Penicillium citrinum with Anti-Cancer and Anti-Inflammatory Activities
Source: Mar Drugs. 2021 Jan 8;19(1):25. doi: 10.3390/md19010025 (PMC7827422; doi:10.3390/md19010025)
Supplement: Supplementary file 1 [file marinedrugs-19-00025-s001.pdf]

Supplementary Materials

Rare Chromone Derivatives from the Marine-Derived *Penicillium citrinum* with Anti-Cancer and Anti-Inflammatory Activities

Yi-Cheng Chu [1], Chun-Hao Chang [2], Hsiang-Ruei Liao [3], Ming-Jen Cheng [4], Ming-Der Wu [4], Shu-Ling Fu [1,*] and Jih-Jung Chen [5,6,*]

[1] Institute of Traditional Medicine, School of Medicine, National Yang-Ming University, Taipei 112, Taiwan; xbox88888@ym.edu.tw (Y.-C.C.)
[2] Institute of Biopharmaceutical Sciences, Pharmaceutical Sciences, National Yang-Ming University, Taipei 112, Taiwan; howard860212@ym.edu.tw (C.-H.C.)
[3] Graduate Institute of Natural Products, College of Medicine, Chang Gung University, Taoyuan 333, Taiwan; liaoch@mail.cgu.edu.tw (H.-R.L.)
[4] Bioresource Collection and Research Center (BCRC), Food Industry Research and Development Institute (FIRDI), Hsinchu 300, Taiwan; cmj@firdi.org.tw (M.-J.C.); wmd@firdi.org.tw (M.-D.W.)
[5] Faculty of Pharmacy, School of Pharmaceutical Sciences, National Yang-Ming University, Taipei 112, Taiwan
[6] Department of Medical Research, China Medical University Hospital, China Medical University, Taichung 404, Taiwan
* Correspondence: chenjj@ym.edu.tw (J.-J.C.); slfu@ym.edu.tw (S.-L.F.)
Tel.: +886-2-2826-7195 (J.-J.C.); +886-2-2826-7177 (S.-L.F.) ; Fax: +886-2-2823-2940 (J.-J.C.); +886-2-2822-5044 (S.-L.F.)

Contents

Figure S1. HRESIMS spectrum of **1**..S4

Figure S2. ^{1}H NMR spectrum ($CDCl_3$, 500 MHz) of **1**...S4

Figure S3. ^{13}C NMR spectrum ($CDCl_3$, 125 MHz) of **1**..S5

Figure S4. ^{1}H-^{1}H COSY spectrum of **1**...S5

Figure S5. Expanded ^{1}H-^{1}H COSY spectrum of **1**...S6

Figure S6. ROESY spectrum of **1**...S6

Figure S7. Expanded ROESY spectrum of **1**...S7

Figure S8. HMBC spectrum of **1**...S7

Figure S9. HSQC spectrum of **1**..S8

Figure S10. CD spectrum of **1**...S8

Figure S11. HRESIMS spectrum of **2**...S9

Figure S12. ^{1}H NMR spectrum ($CDCl_3$, 500 MHz) of **2**..S9

Figure S13. ^{13}C NMR spectrum ($CDCl_3$, 125 MHz) of **2**...S10

Figure S14. ^{1}H-^{1}H COSY spectrum of **2**...S10

Figure S15. Expanded ^{1}H-^{1}H COSY spectrum of **2**...S11

Figure S16. ROESY spectrum of **2**..S11

Figure S17. Expanded ROESY spectrum of **2**...S12

Figure S18. HMBC spectrum of **2**...S12

Figure S19. HSQC spectrum of **2**..S13

Figure S20. CD spectrum of **2**..S13

Figure S21. HRESIMS spectrum of **3**..S14

Figure S22. ^{1}H NMR spectrum ($CDCl_3$, 500 MHz) of **3**...S14

Figure S23. ^{13}C NMR spectrum ($CDCl_3$, 125 MHz) of **3**...S15

Figure S24. ^{1}H-^{1}H COSY spectrum of **3**...S15

Figure S25. Expanded ^{1}H-^{1}H COSY spectrum of **3**...S16

Figure S26. ROESY spectrum of **3**..S16

Figure S27. Expanded ROESY spectrum of **3**...S17

Figure S28. HMBC spectrum of **3**........ S17
Figure S29. HSQC spectrum of **3**........ S18
Figure S30. CD spectrum of **3**........ S18
Figure S31. HRESIMS spectrum of **4**........ S19
Figure S32. ^{1}H NMR spectrum ($CDCl_3$, 500 MHz) of **4**........ S19
Figure S33. ^{13}C NMR spectrum ($CDCl_3$, 125 MHz) of **4**........ S20
Figure S34. CD spectrum of **4**........ S20
Figure S35. HRESIMS spectrum of **5**........ S21
Figure S36. ^{1}H NMR spectrum ($CDCl_3$, 600 MHz) of **5**........ S21
Figure S37. HRESIMS spectrum of **6**........ S22
Figure S38. ^{1}H NMR spectrum ($CDCl_3$, 600 MHz) of **6**........ S22
Figure S39. HRESIMS spectrum of **7**........ S23
Figure S40. ^{1}H NMR spectrum ($CDCl_3$, 600 MHz) of **7**........ S23
Figure S41. CD and ECD spectra of epiremisporine B........ S24
Table S1. ^{1}H NMR spectrum of **4** and epiremisporine B........ S24
Table S2. ^{13}C NMR spectrum of **4** and epiremisporine B........ S25
Table S3. The ROESY correlations for compounds **1–3**........ S26

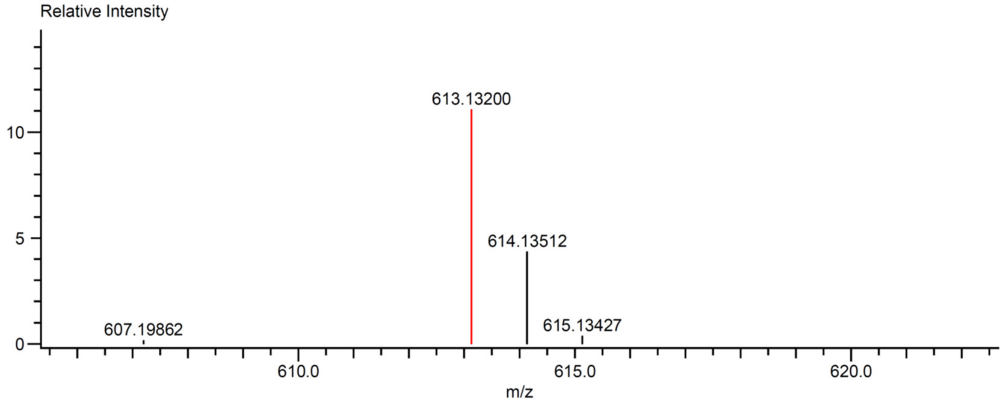

Mass	Intensity	Calc. Mass	Mass Difference [mDa]	Mass Difference [ppm]	Possible Formula
613.13200	5276.54	613.13219	-0.20	-0.32	$^{12}C_{31}{}^{1}H_{26}{}^{23}Na_{1}{}^{16}O_{12}$

Figure S1. HRESIMS spectrum of 1.

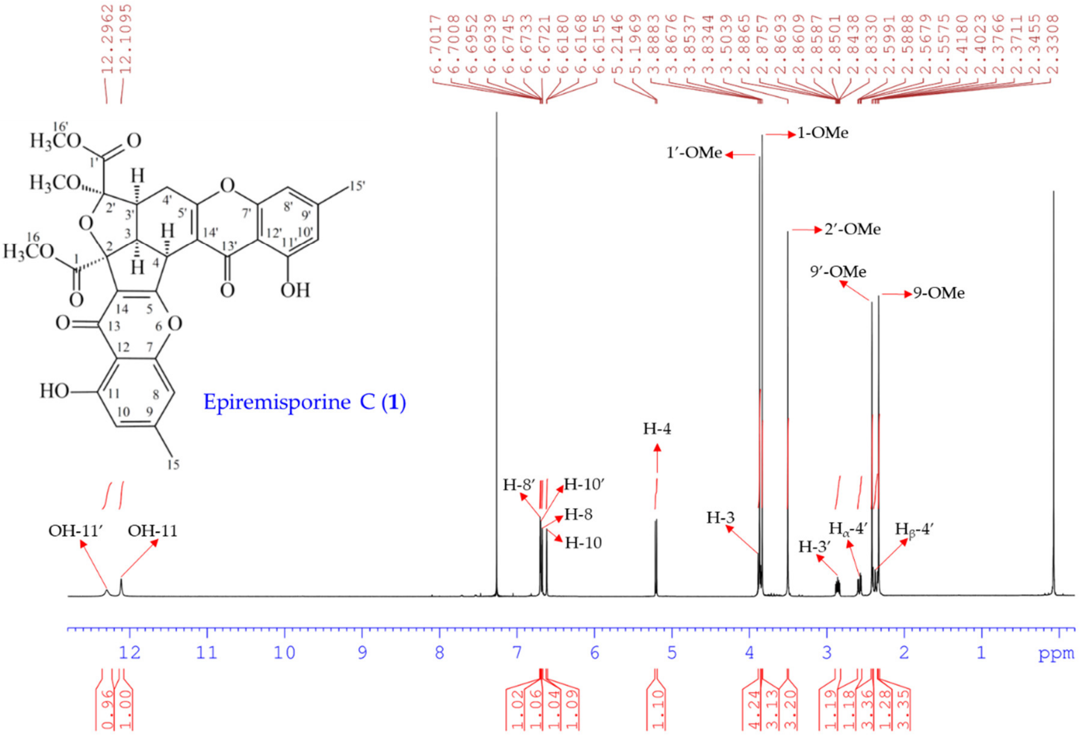

Figure S2. ^{1}H NMR spectrum ($CDCl_3$, 500 MHz) of 1.

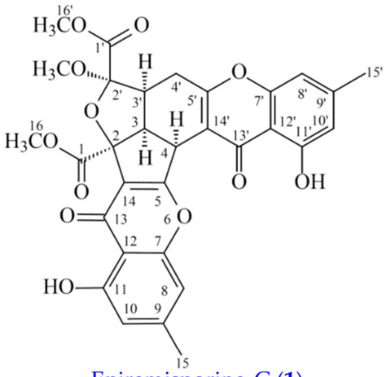

Epiremisporine C (**1**)

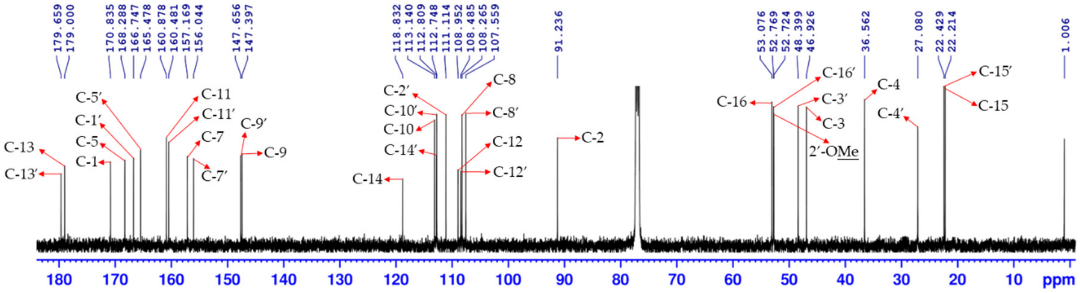

Figure S3. ^{13}**C NMR (CDCl**$_3$**, 125 MHz) spectrum of 1.**

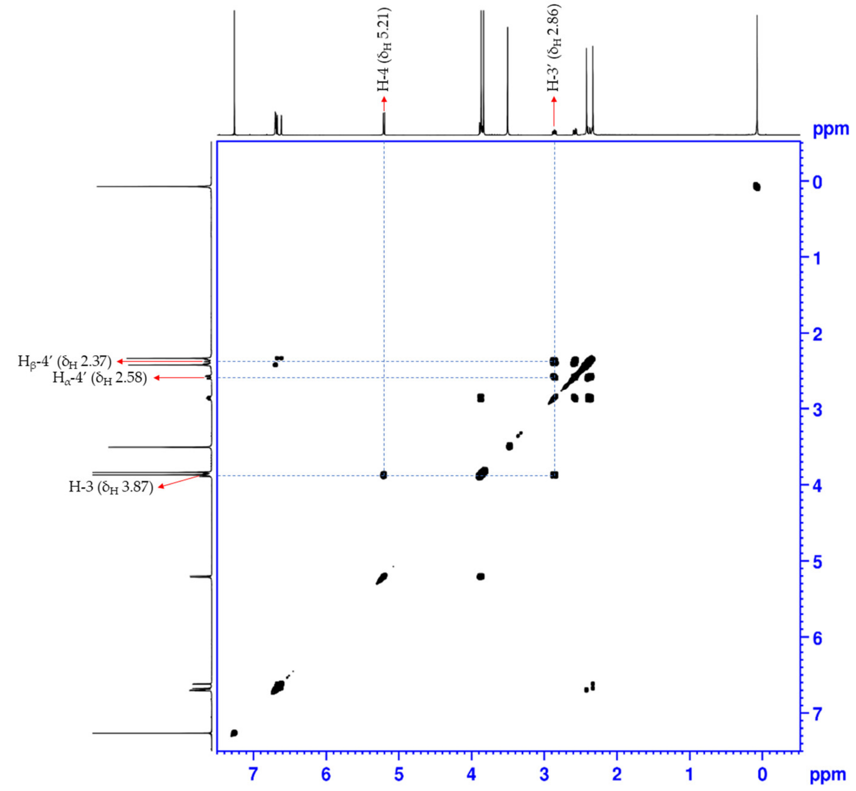

Figure S4. ^{1}**H–**^{1}**H COSY spectrum of 1.**

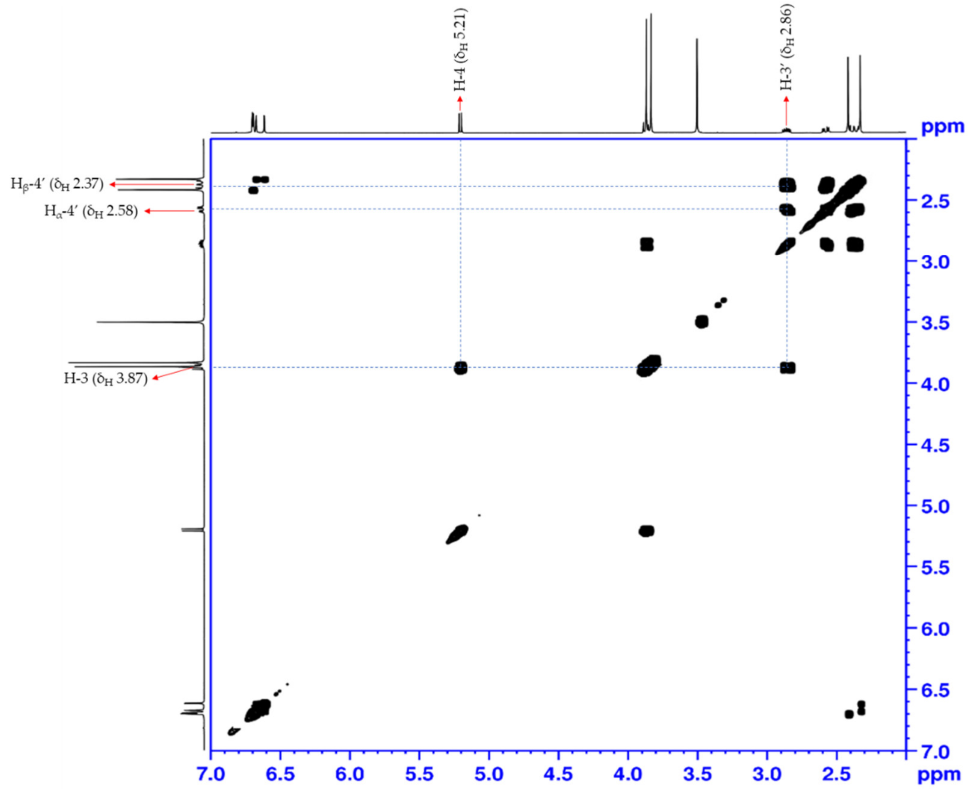

Figure S5. Expanded ^{1}H-^{1}H COSY spectrum of 1.

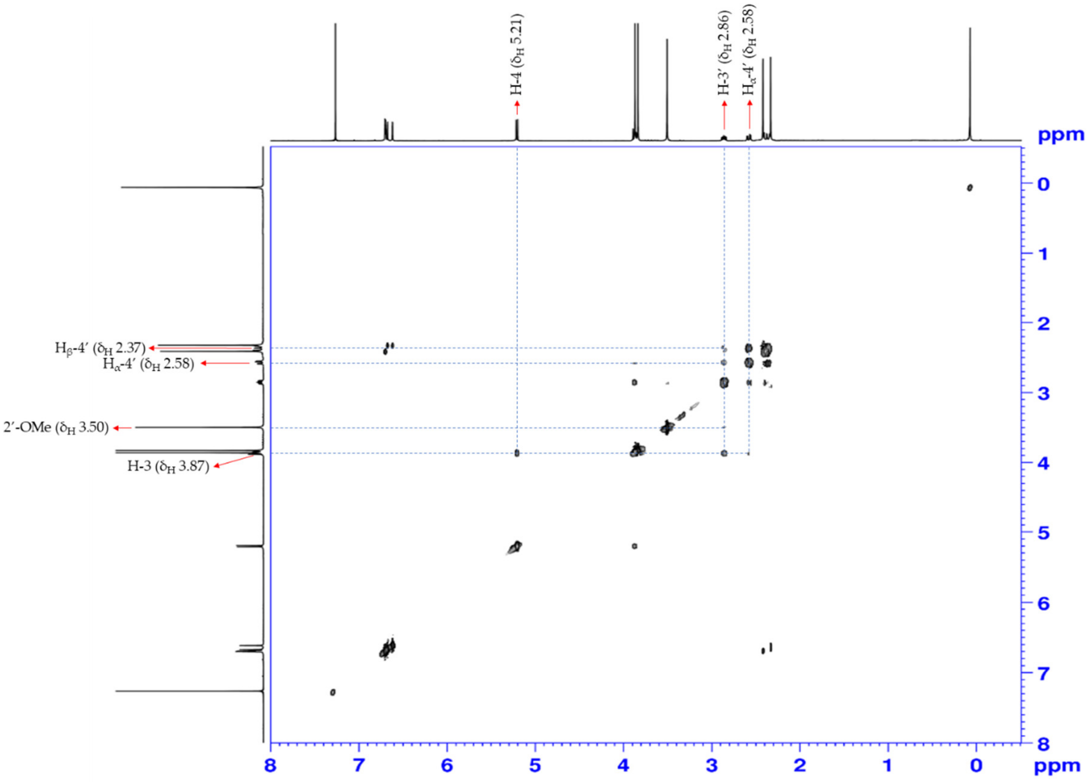

Figure S6. ROESY spectrum of 1.

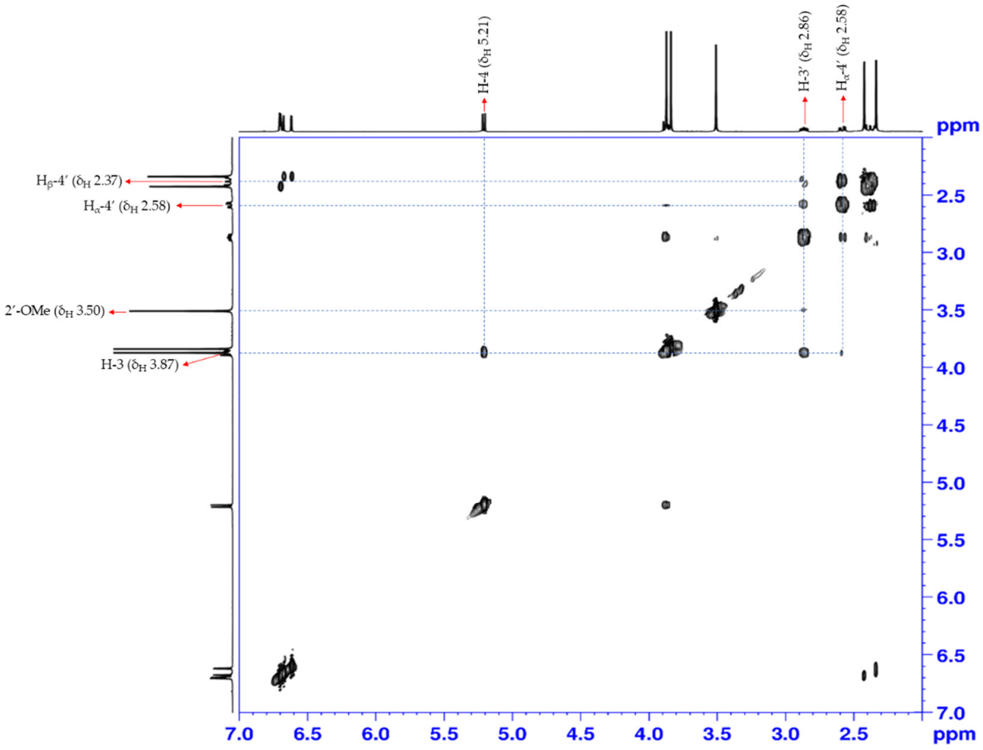

Figure S7. Expanded ROESY spectrum of 1.

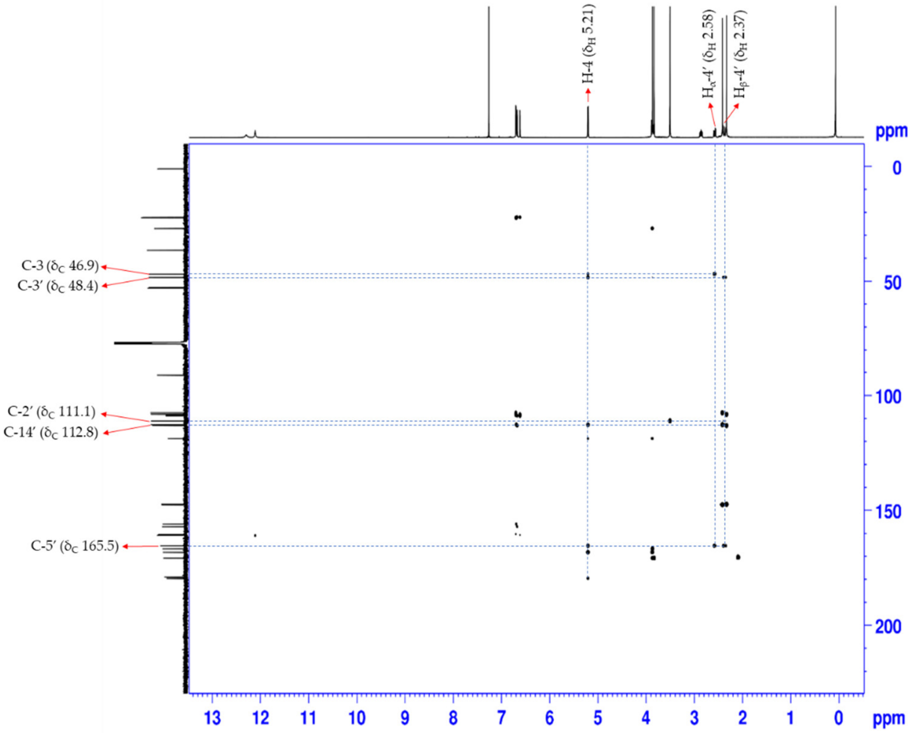

Figure S8. HMBC spectrum of 1.

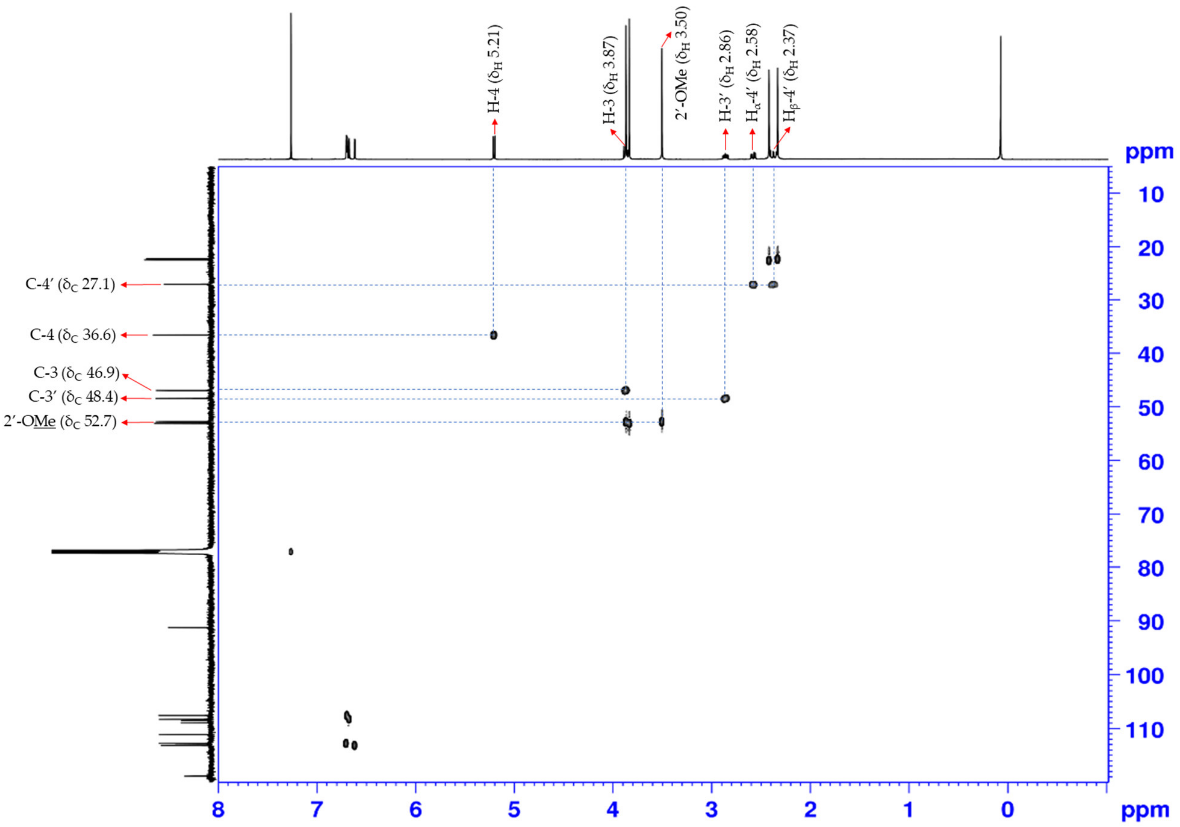

Figure S9. HSQC spectrum of 1.

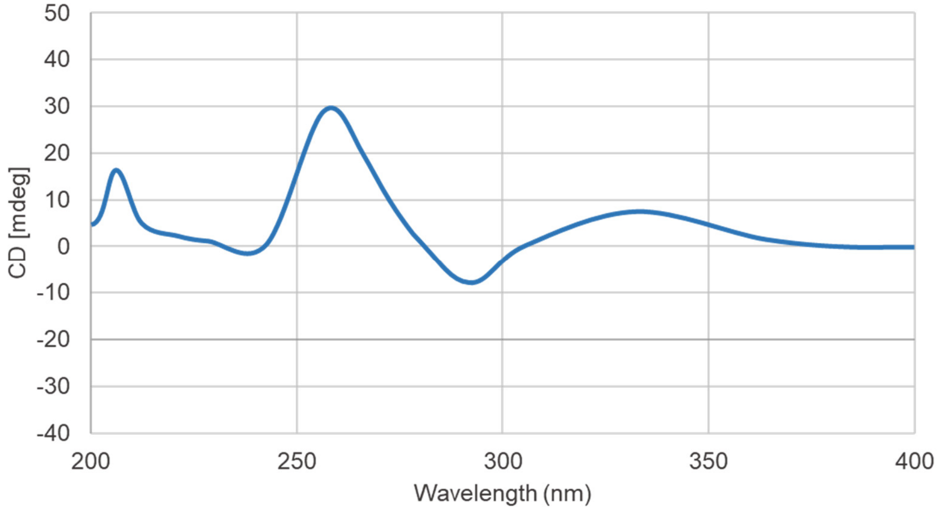

Figure S10. CD spectrum of 1.

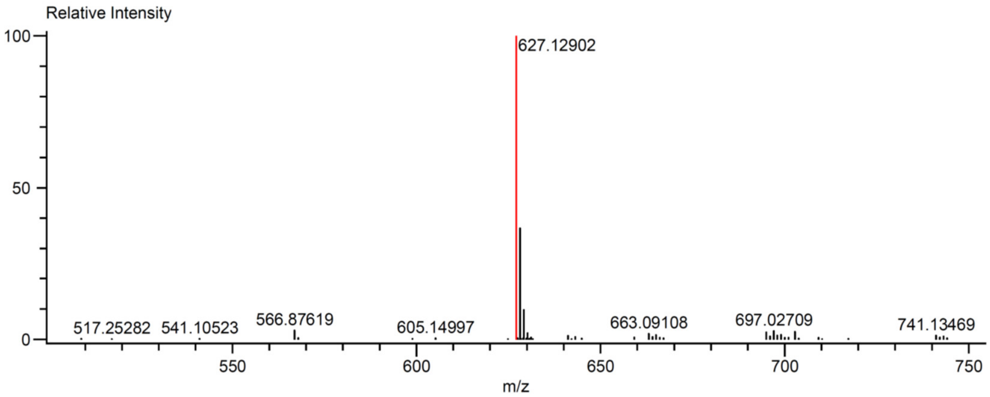

Mass	Intensity	Calc. Mass	Mass Difference [mDa]	Mass Difference [ppm]	Possible Formula
627.12902	103212.93	627.14784	-18.83	-30.02	$^{12}C_{32}{}^{1}H_{28}{}^{23}Na_{1}{}^{16}O_{12}$

Figure S11. HRESIMS spectrum of 2.

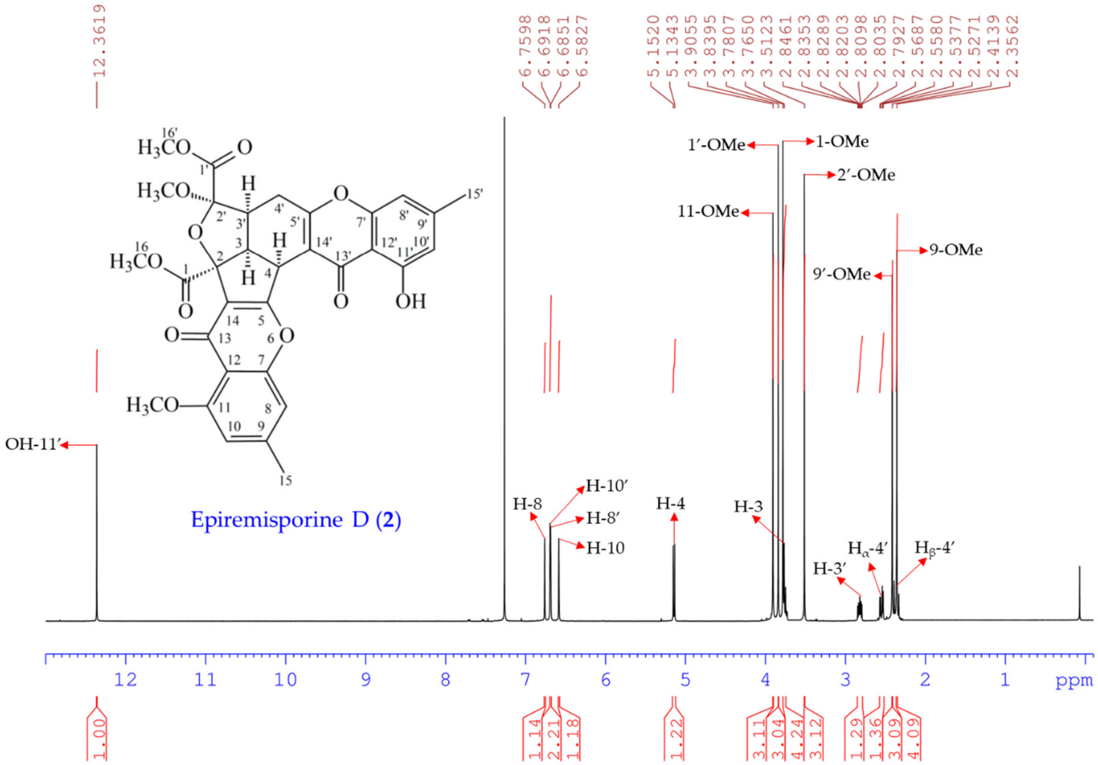

Figure S12. ^{1}H NMR spectrum ($CDCl_3$, 500 MHz) of 2.

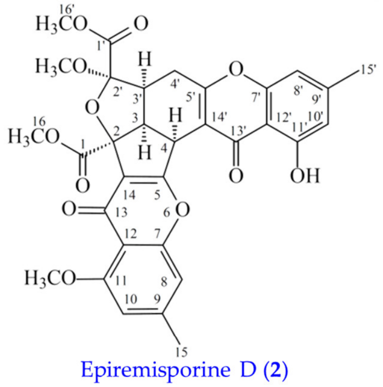

Epiremisporine D (**2**)

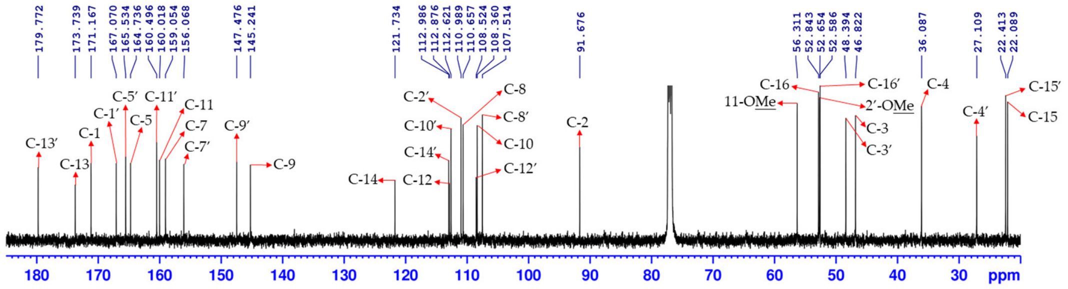

Figure S13. ^{13}**C NMR spectrum (CDCl**$_3$**, 125 MHz) of 2.**

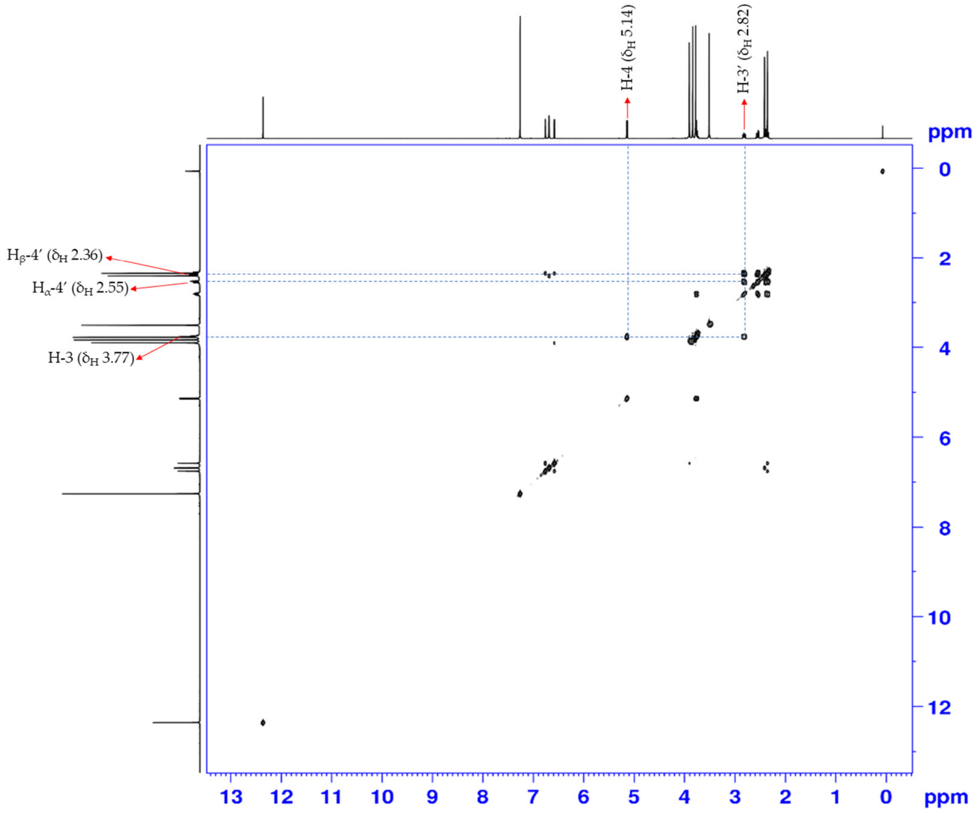

Figure S14. ^{1}**H–**^{1}**H COSY spectrum of 2.**

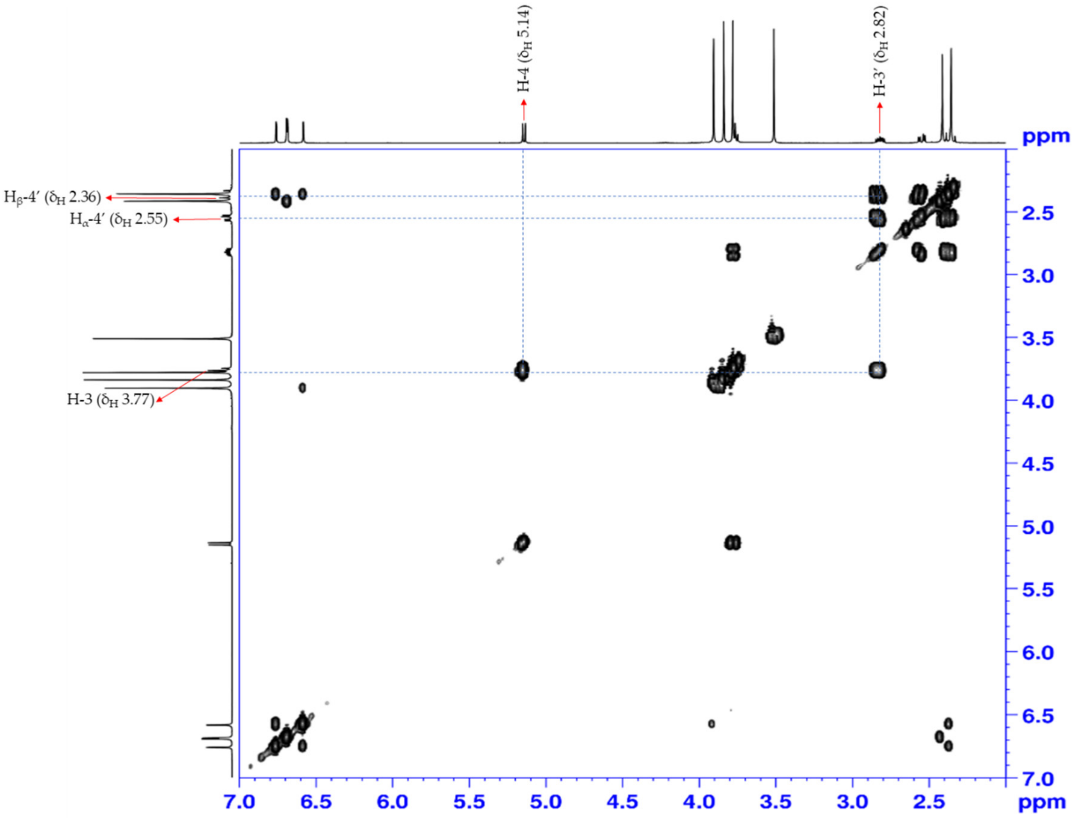

Figure S15. Expanded ¹H–¹H COSY spectrum of 2.

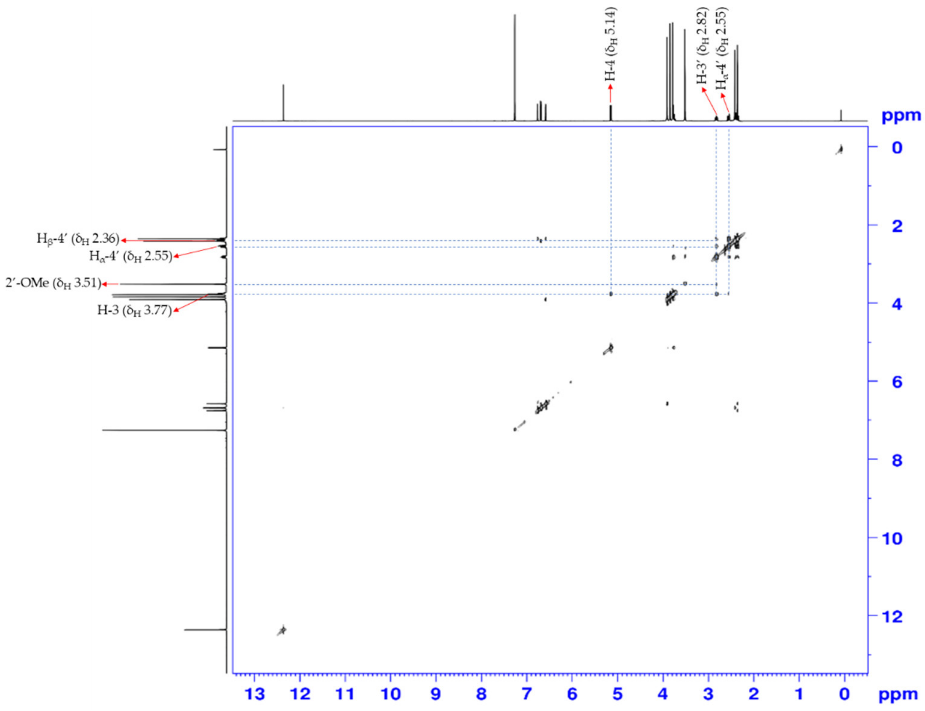

Figure S16. ROESY spectrum of 2.

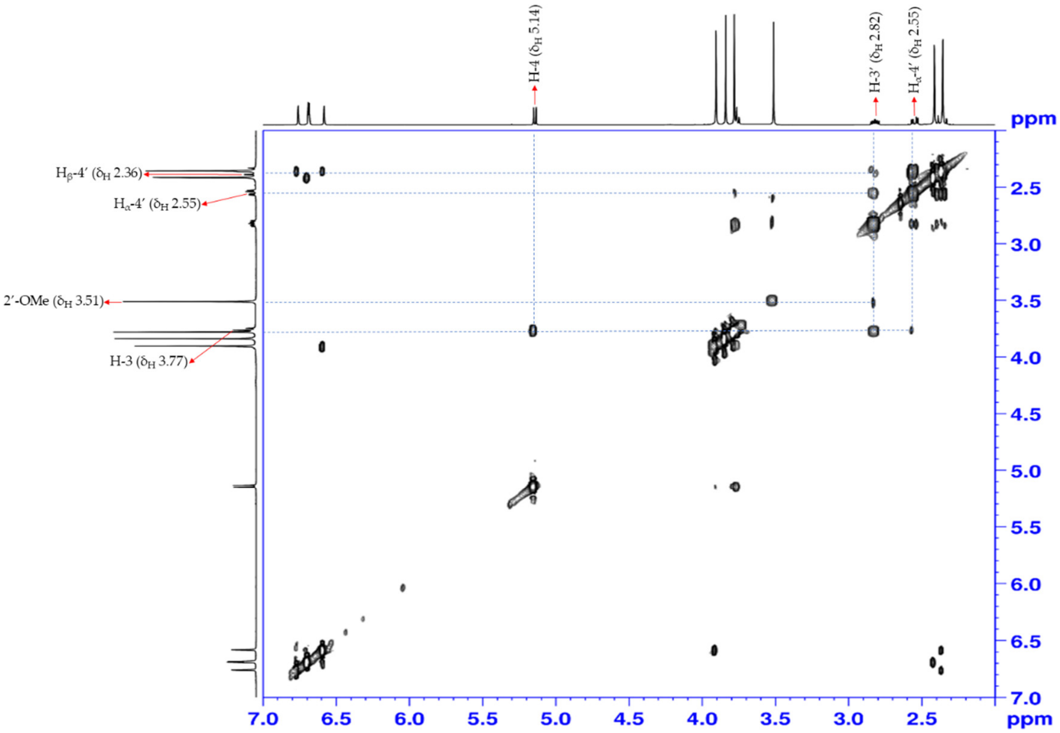

Figure S17. Expanded ROESY spectrum of 2.

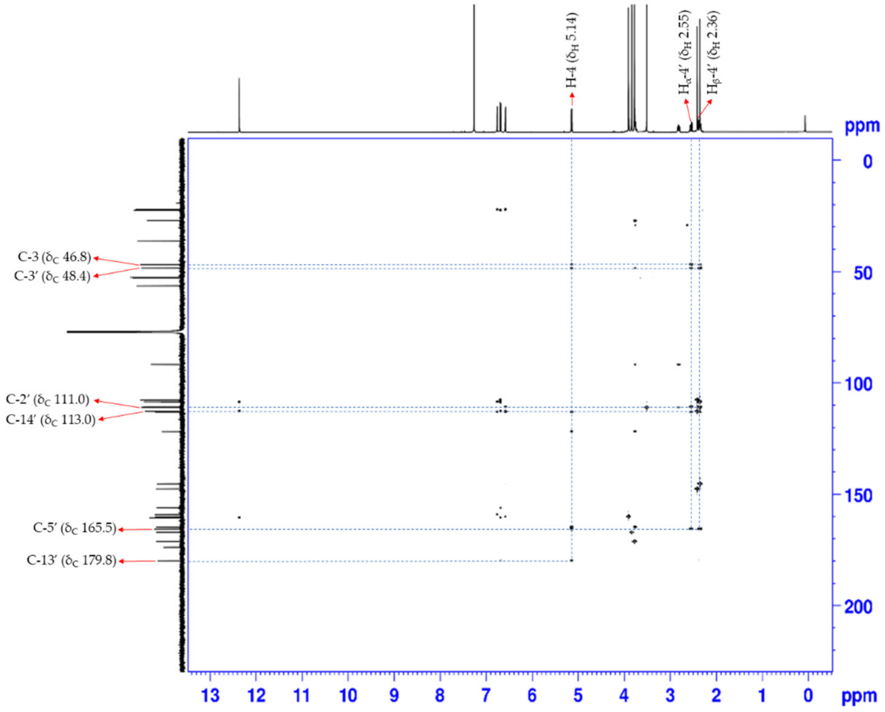

Figure S18. HMBC spectrum of 2.

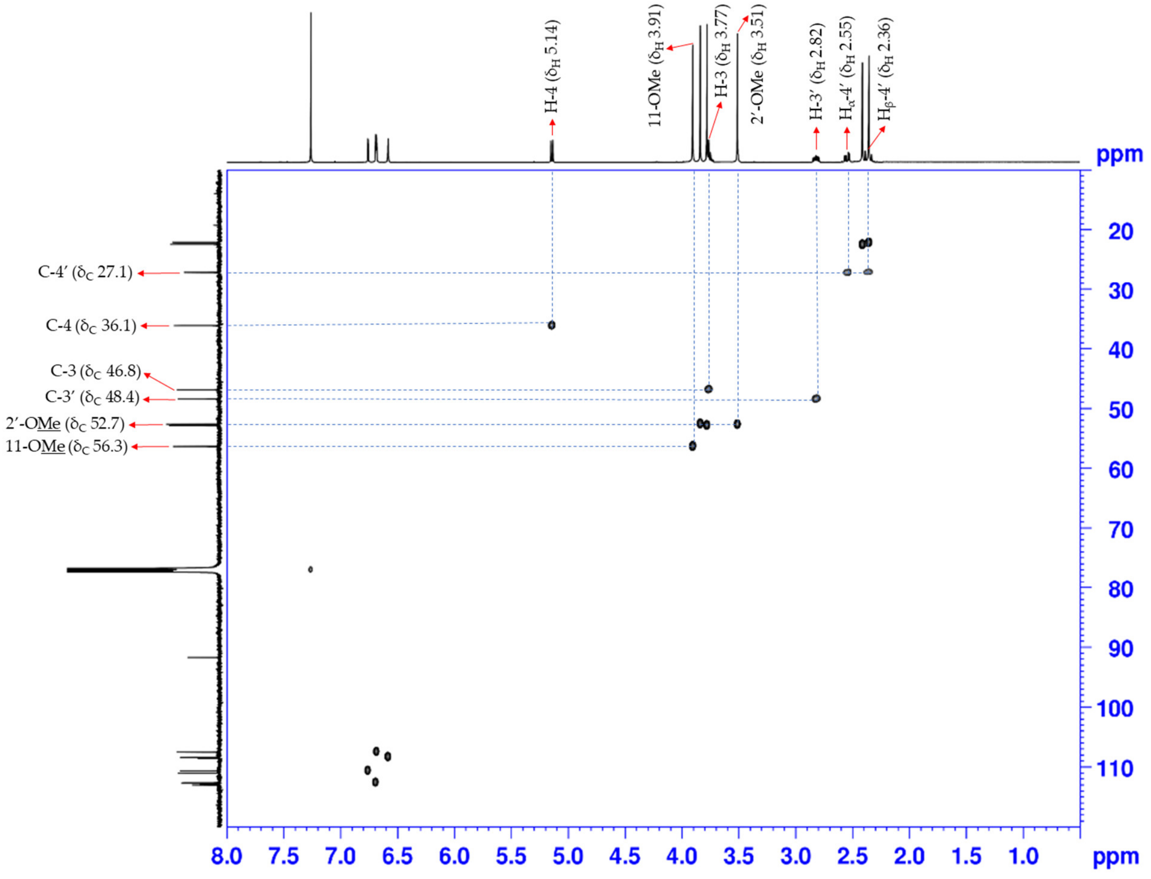

Figure S19. HSQC spectrum of 2.

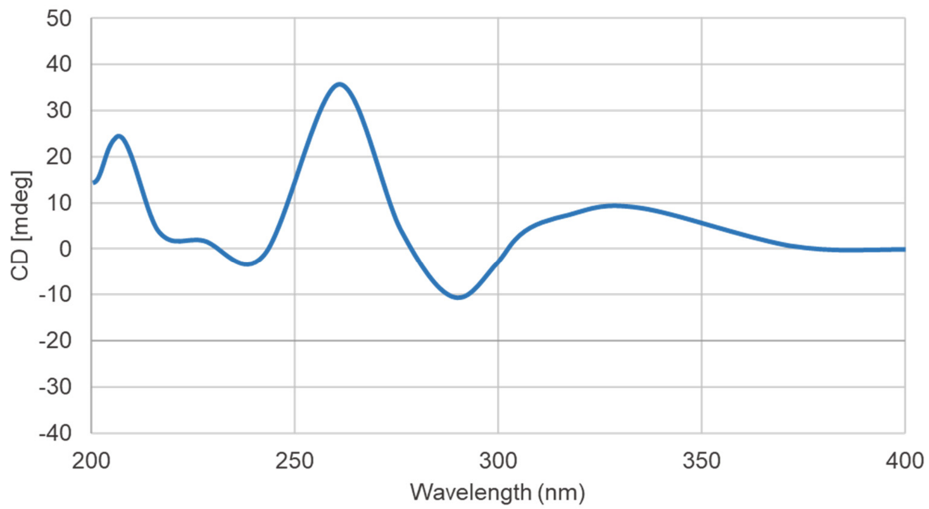

Figure S20. CD spectrum of 2.

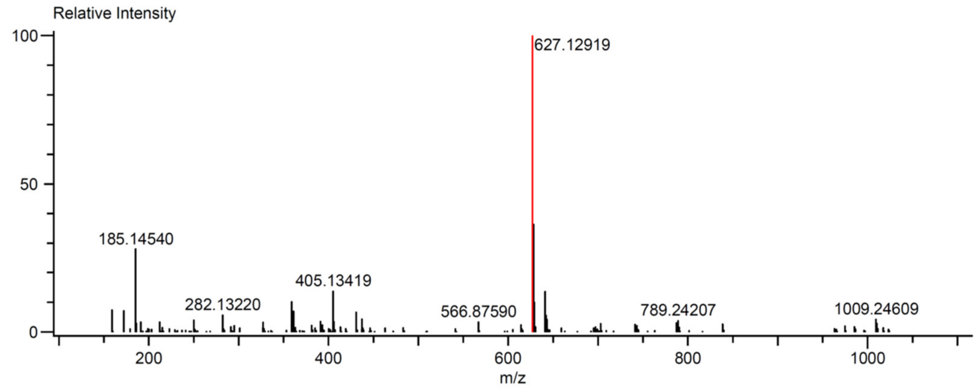

Mass	Intensity	Calc. Mass	Mass Difference [mDa]	Mass Difference [ppm]	Possible Formula
627.12919	35807.43	627.14784	-18.66	-29.75	$^{12}C_{32}{}^{1}H_{28}{}^{23}Na_{1}{}^{16}O_{12}$

Figure S21. HRESIMS spectrum of 3.

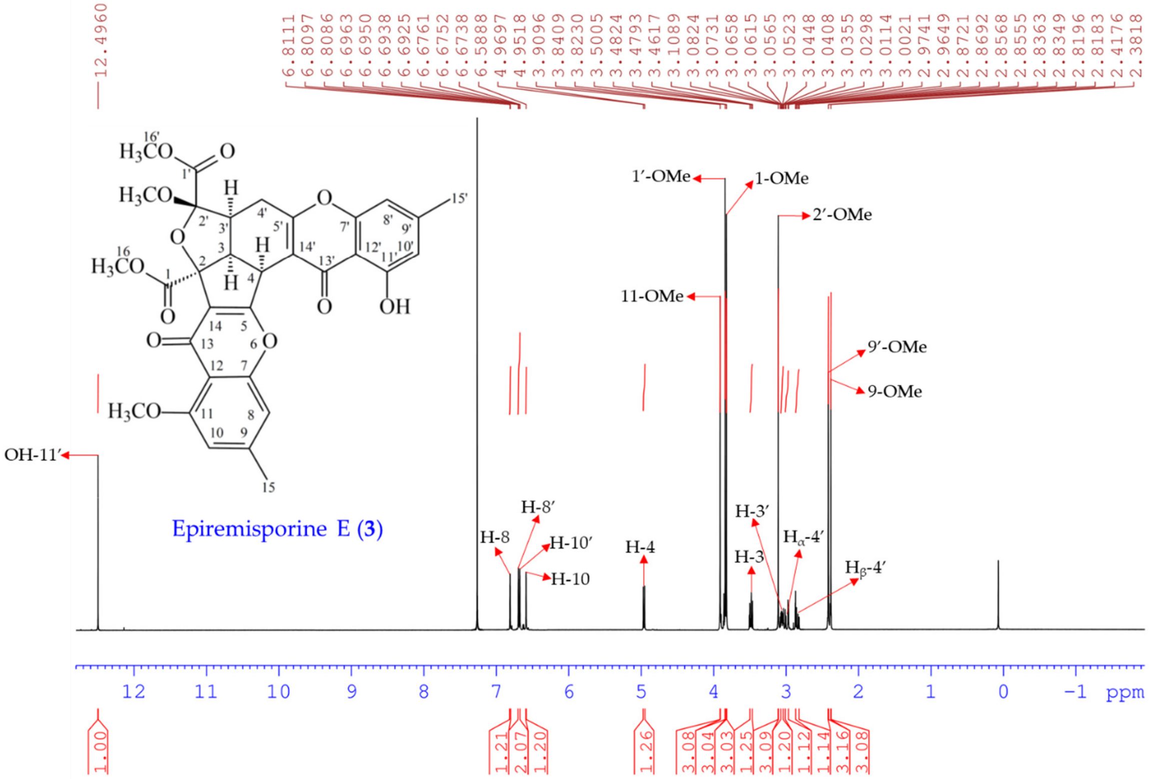

Figure S22. ^{1}H NMR spectrum ($CDCl_3$, 500 MHz) of 3.

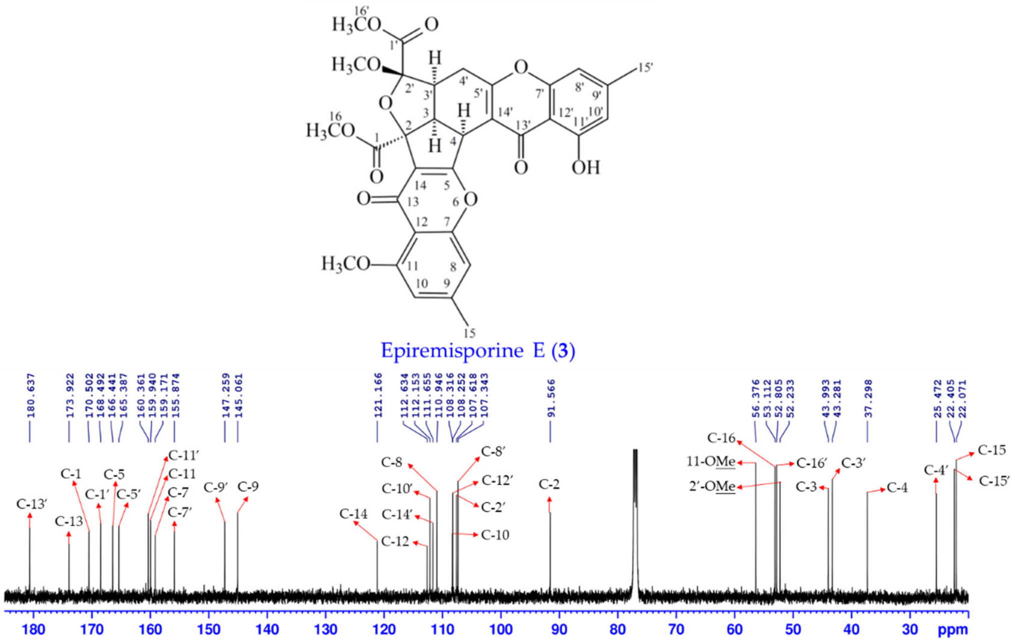

Figure S23. ^{13}**C NMR spectrum of 3 (CDCl**$_3$**, 125 MHz) of 3.**

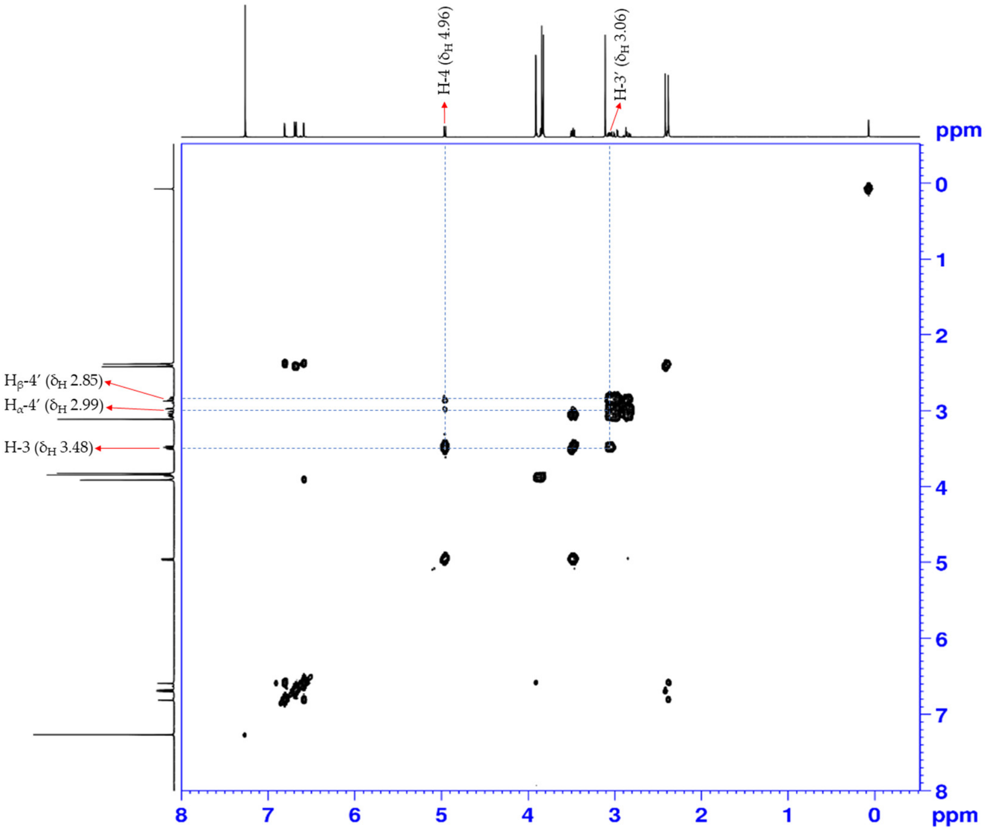

Figure S24. ^{1}**H–**^{1}**H COSY spectrum of 3.**

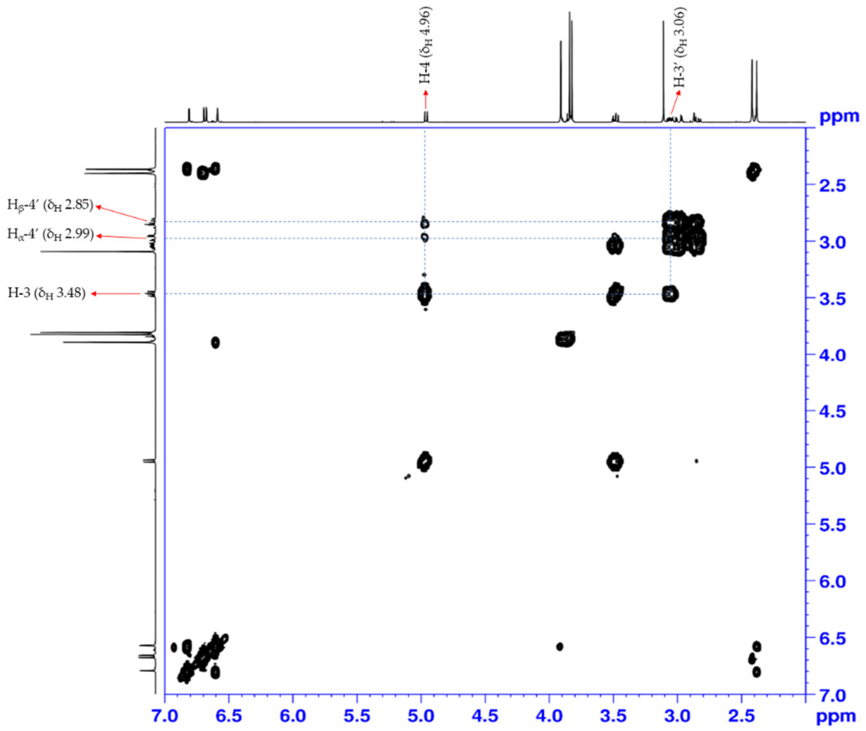

Figure S25. Expanded ^{1}H–^{1}H COSY spectrum of 3.

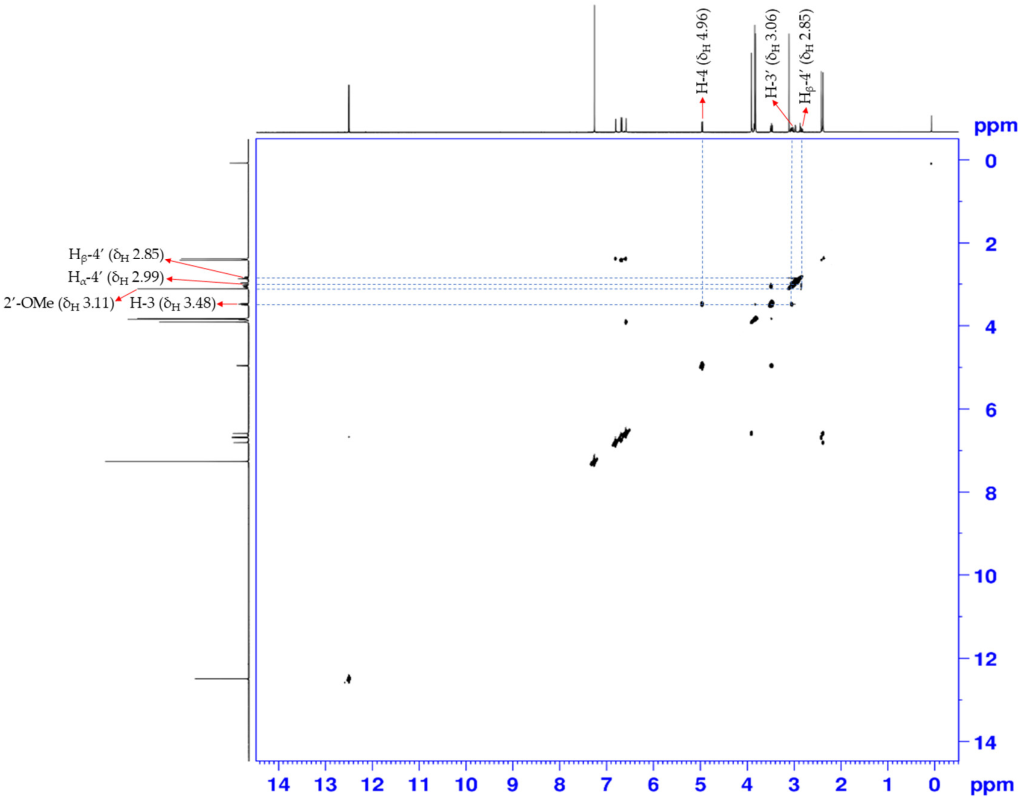

Figure S26. ROESY spectrum of 3.

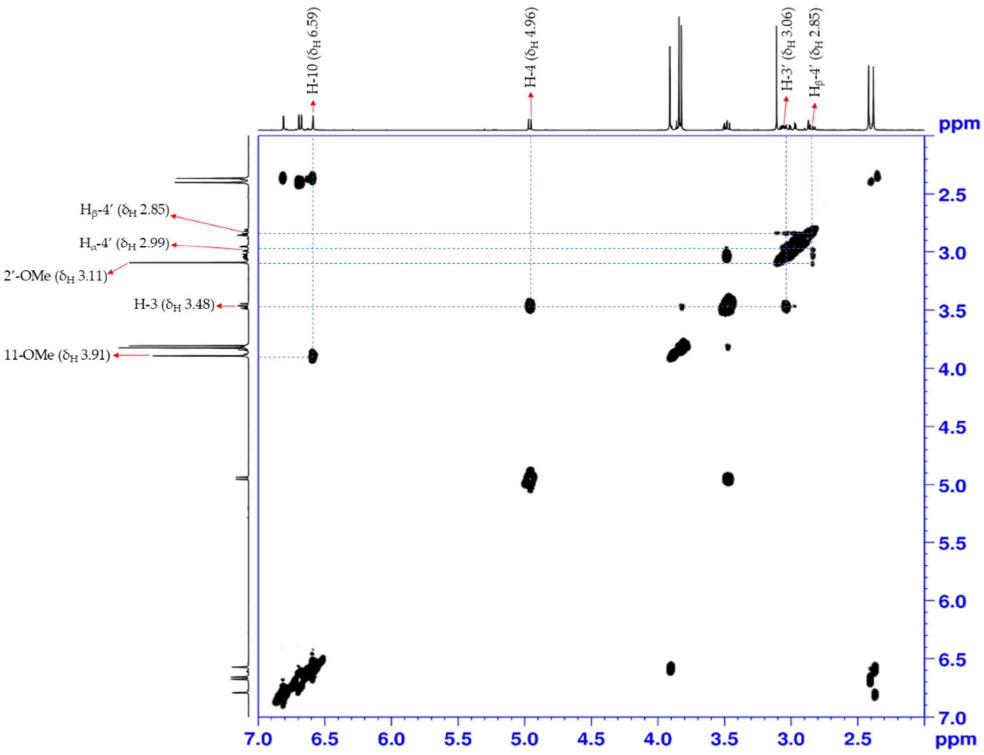

Figure S27. Expanded ROESY spectrum of 3.

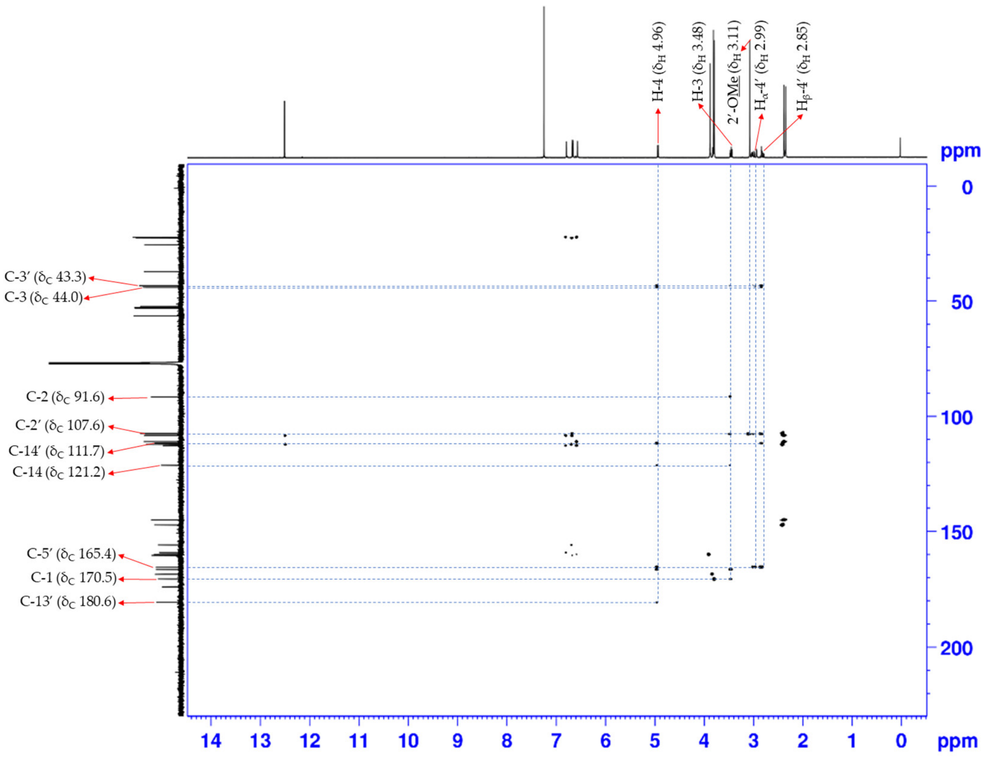

Figure S28. HMBC spectrum of 3.

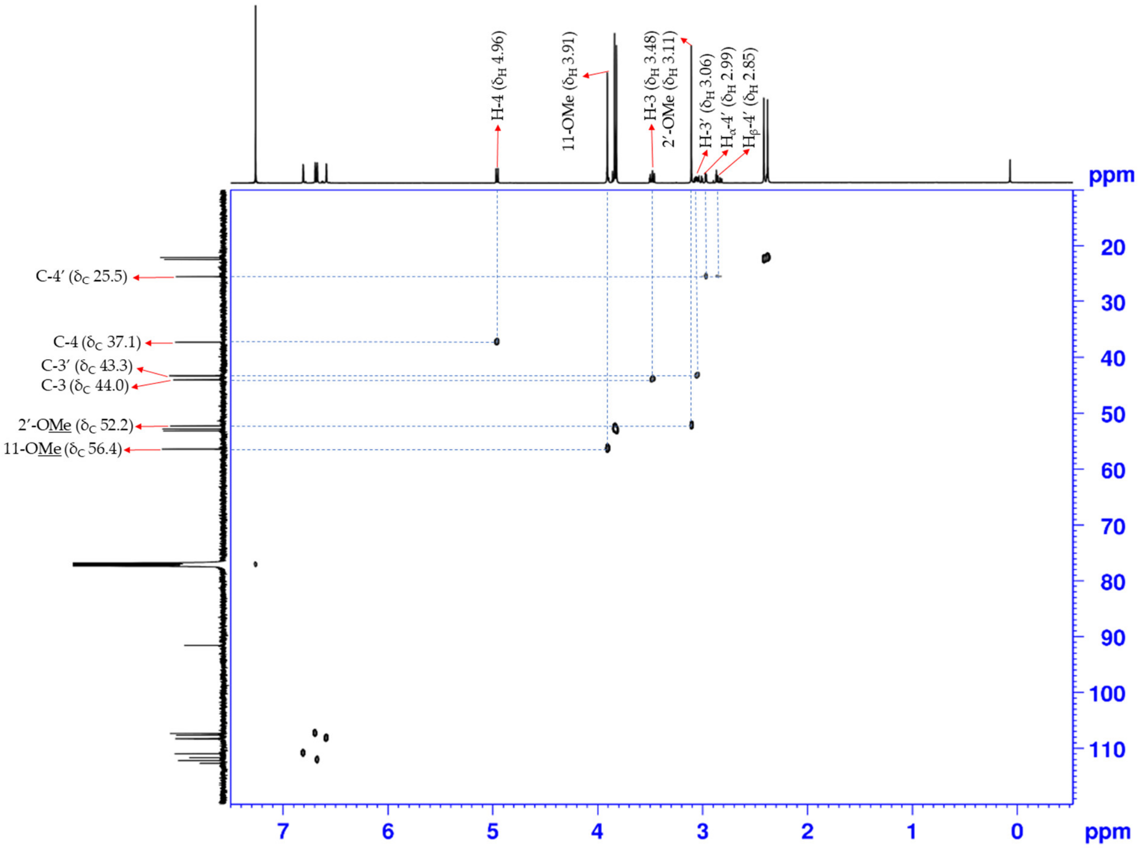

Figure S29. HSQC spectrum of 3.

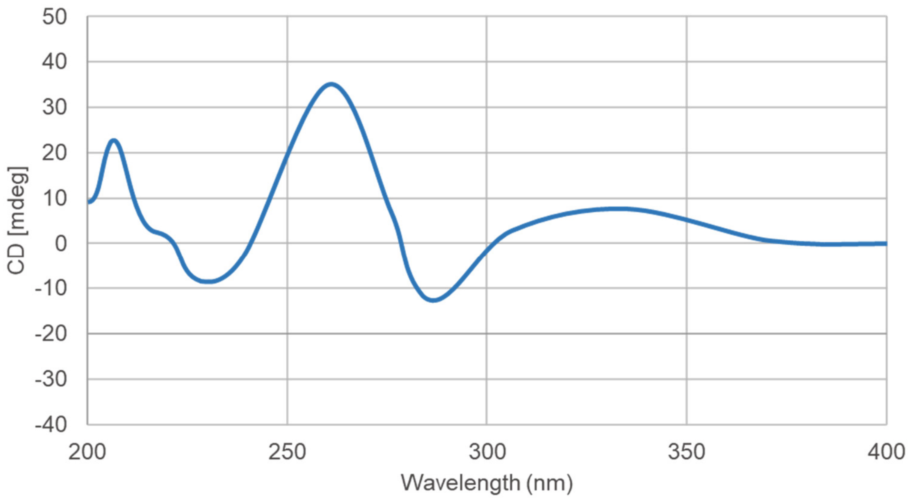

Figure S30. CD spectrum of 3.

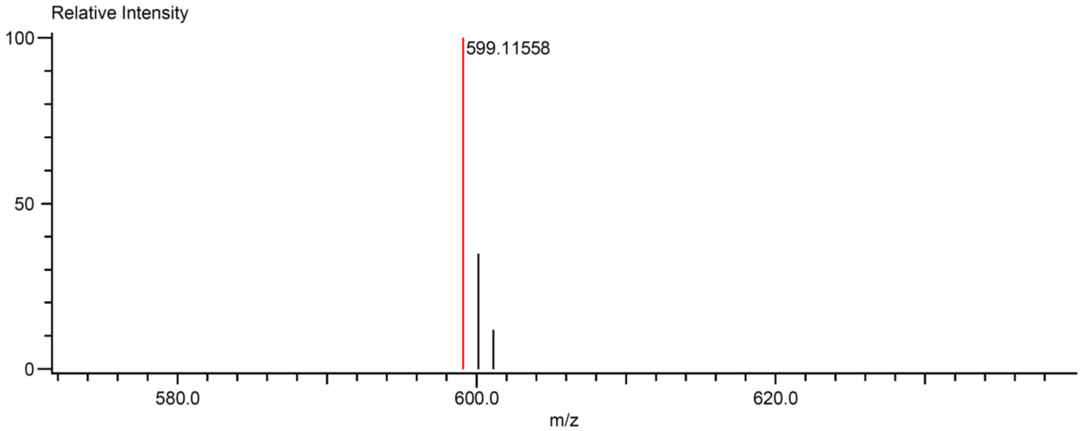

Mass	Intensity	Calc. Mass	Mass Difference [mDa]	Mass Difference [ppm]	Possible Formula
599.11558	16867.59	599.11654	-0.96	-1.60	$^{12}C_{30}{}^{1}H_{24}{}^{23}Na_{1}{}^{16}O_{12}$

Figure S31. HRESIMS spectrum of 4

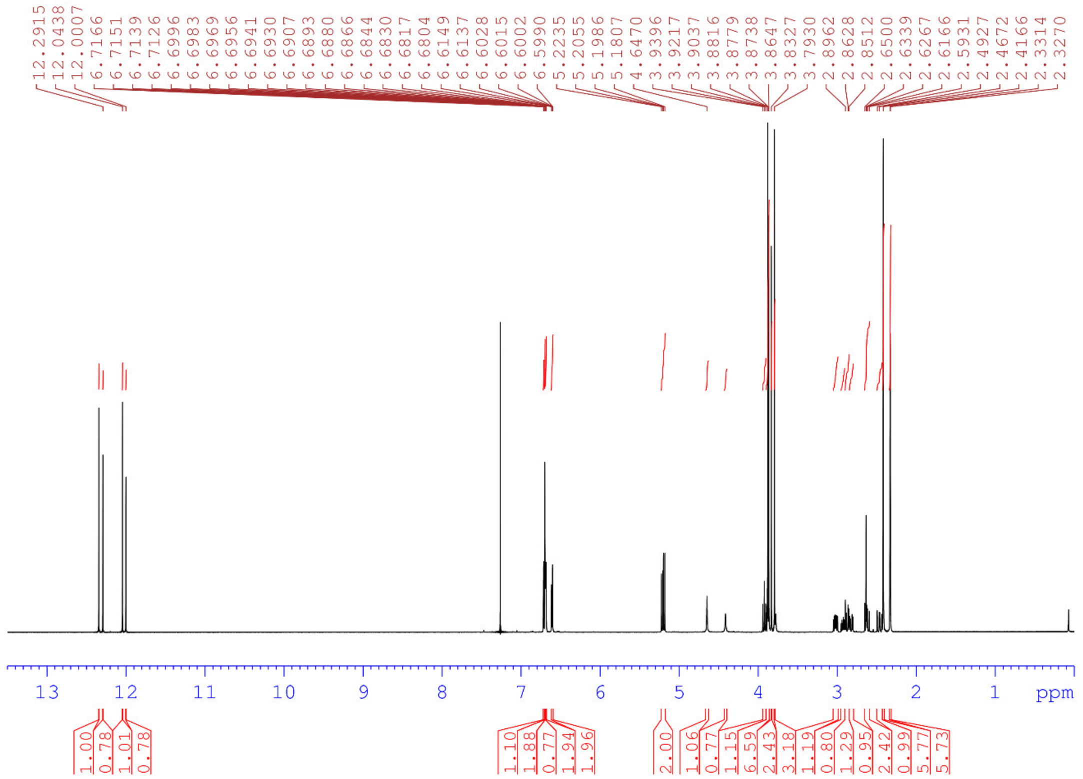

Figure S32. ^{1}H NMR spectrum ($CDCl_3$, 500 MHz) of 4.

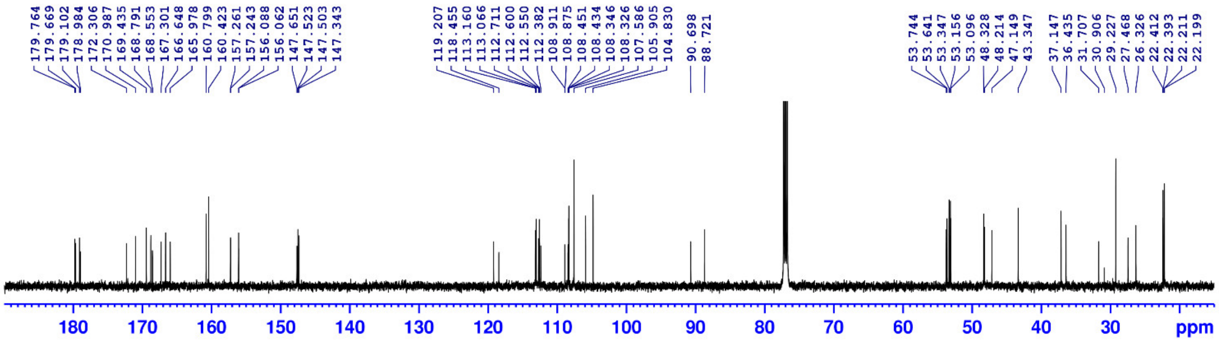

Figure S33. **^{13}C NMR spectrum ($CDCl_3$, 125 MHz) of 4.**

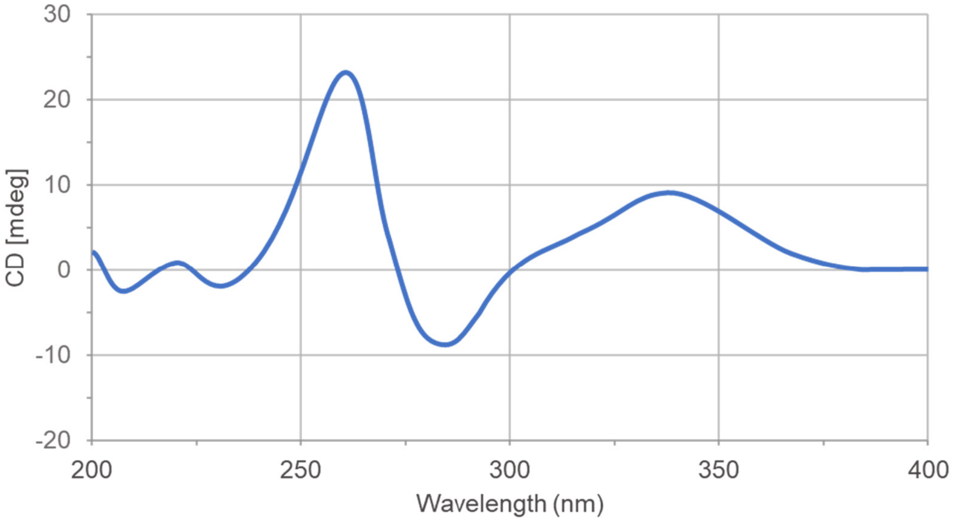

Figure S34. CD spectrum of 4.

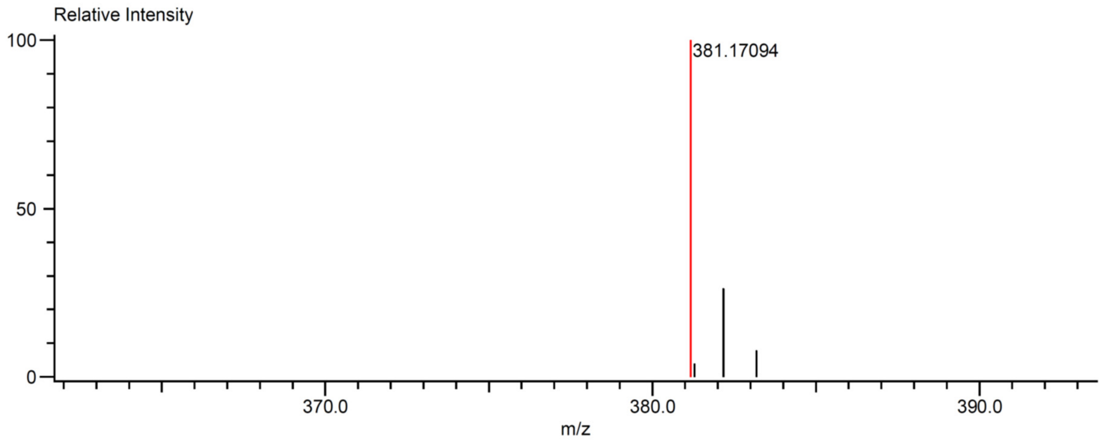

Mass	Intensity	Calc. Mass	Mass Difference [mDa]	Mass Difference [ppm]	Possible Formula
381.17094	17911.35	381.17020	0.74	1.94	$^{12}C_{23}{}^{1}H_{25}{}^{16}O_5$

Figure S35. HRESIMS spectrum of 5.

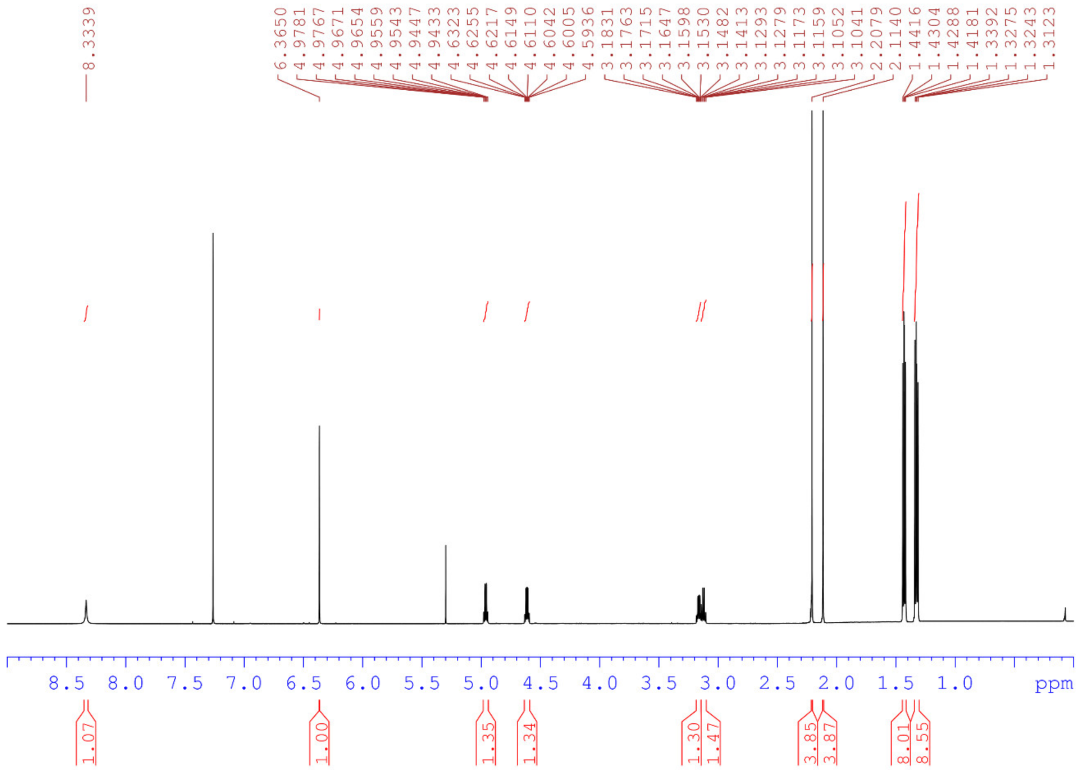

Figure S36. ^{1}H NMR spectrum ($CDCl_3$, 600 MHz) of 5.

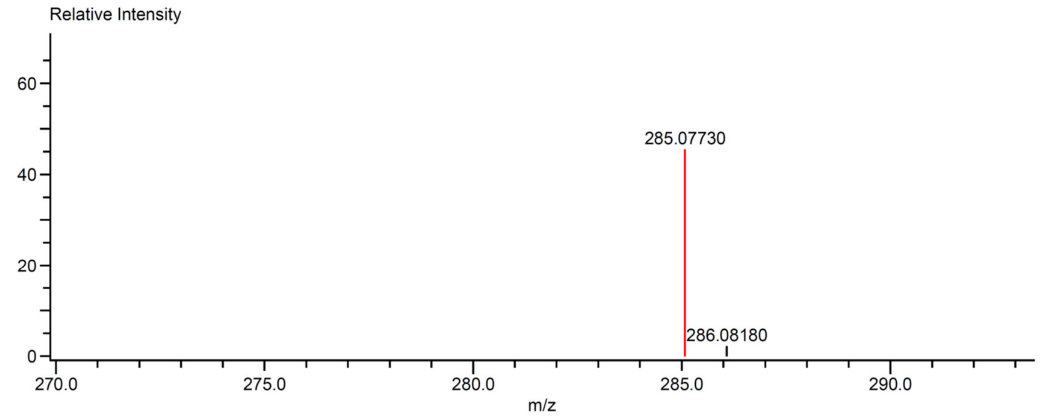

Mass	Intensity	Calc. Mass	Mass Difference [mDa]	Mass Difference [ppm]	Possible Formula
285.07730	2445.10	285.07630	1.00	3.51	$^{12}C_{16}{}^{1}H_{13}{}^{16}O_5$

Figure S37. HRESIMS spectrum of 6.

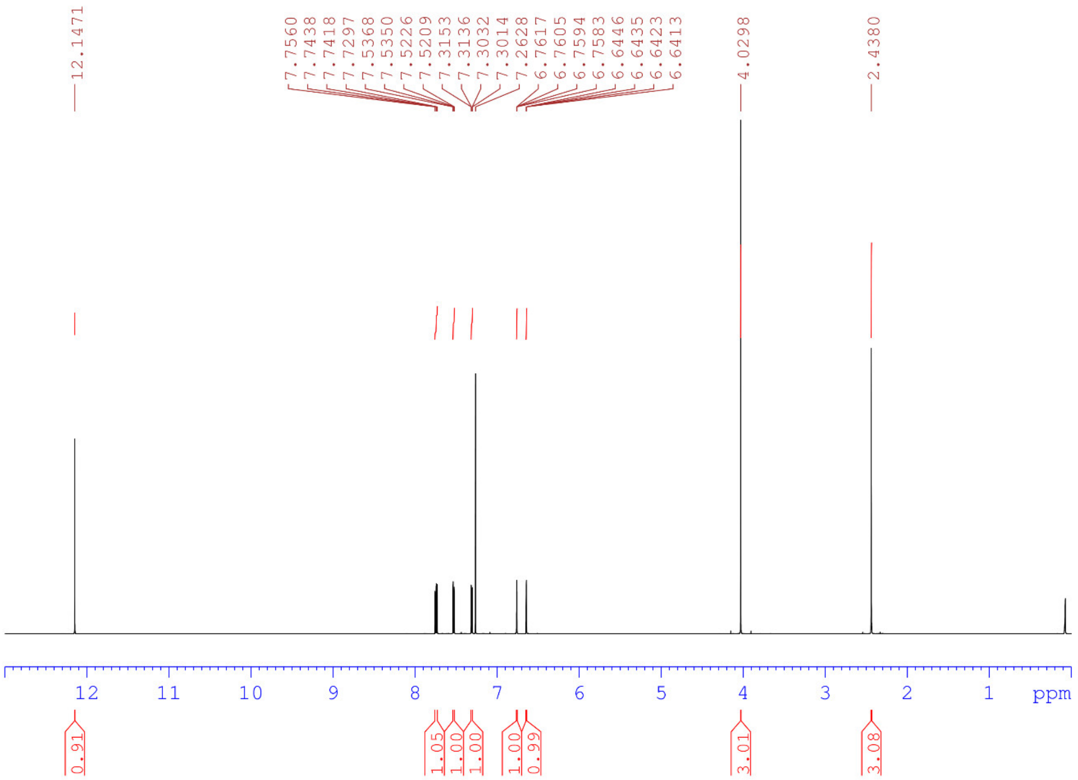

Figure S38. ^{1}H NMR spectrum ($CDCl_3$, 600 MHz) of 6.

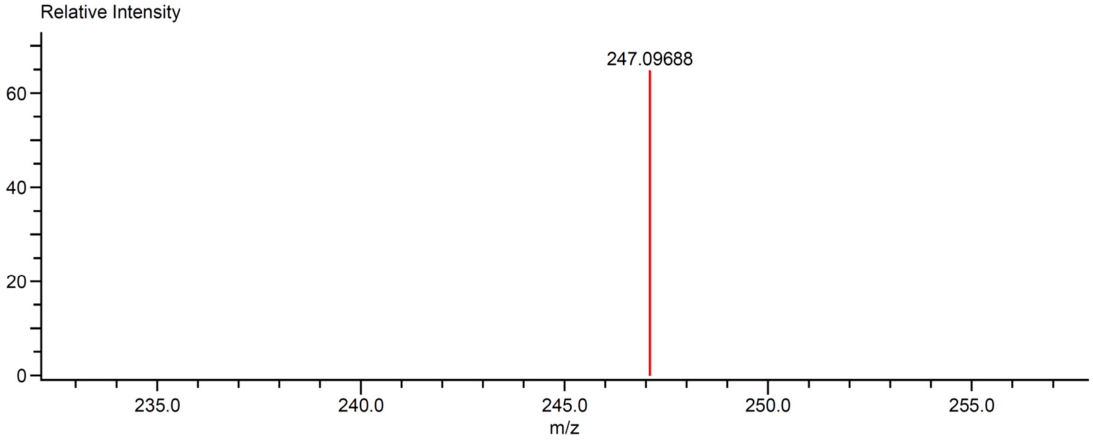

Mass	Intensity	Calc. Mass	Mass Difference [mDa]	Mass Difference [ppm]	Possible Formula
247.09688	2701.97	247.09703	-0.16	-0.64	$^{12}C_{14}{}^{1}H_{15}{}^{16}O_4$

Figure S39. HRESIMS spectrum of 7.

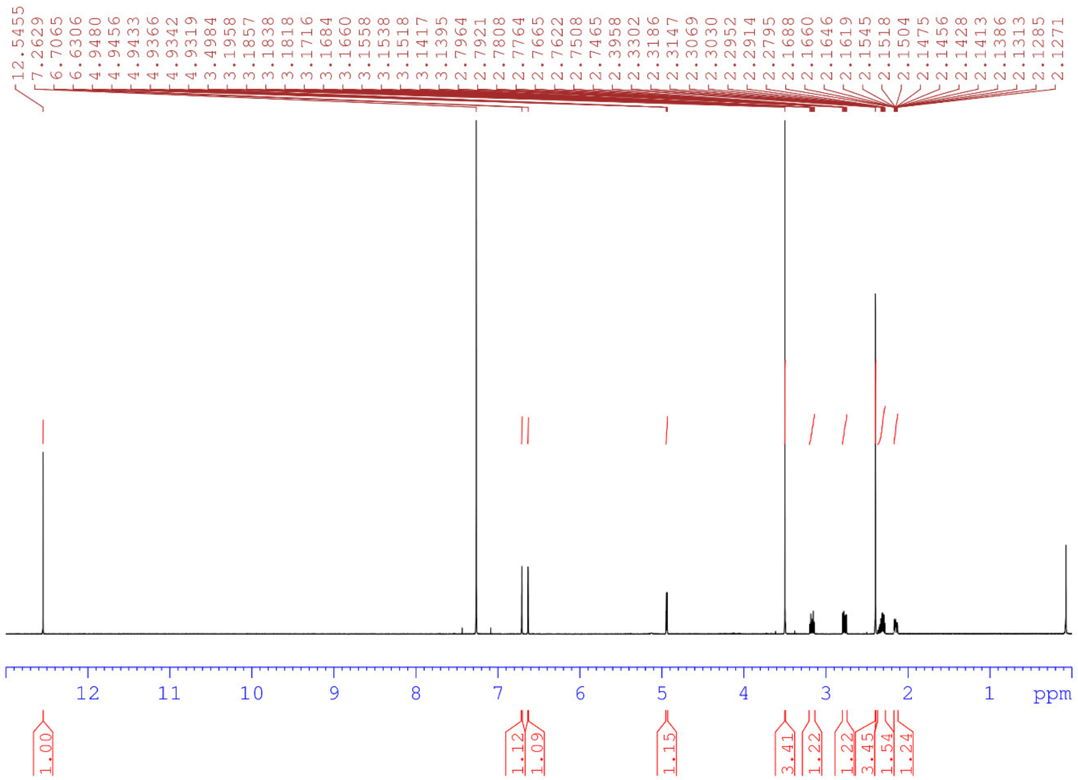

Figure S40. ^{1}H NMR spectrum ($CDCl_3$, 600 MHz) of 7.

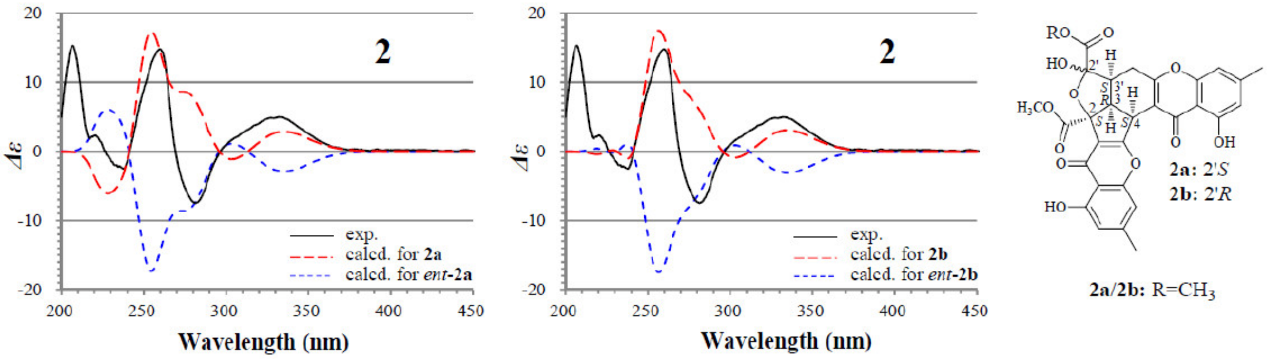

Figure S41. CD and ECD spectra of epiremisporine B.

Table S1. ^{1}H NMR spectrum of 4 and epiremisporine B.

Position	4 (2′*S*) [a]	4 (2′*R*) [a]	epiremisporine B (2′*S*) [b]	epiremisporine B (2′*R*) [b]
	δH (*J* in Hz)			
3	3.92 (t, 9.0)	3.88 (dd, 9.0, 8.3)	3.78 (dd, 9.2, 9.0)	3.88 (dd, 9.0, 8.4)
4	5.19 (d, 9.0)	5.21 (d, 9.0)	4.98 (d, 9.0)	5.02 (d, 9.0)
8	6.68 (br s)	6.69 (br s)	6.79 (s)	6.77 (s)
10	6.60 (br s)	6.61 (br s)	6.64 (s)	6.64 (s)
15	2.33 (s)	2.33 (s)	2.30 (s)	2.28 (s)
16	3.79 (s)	3.83 (s)	3.69 (s)	3.70 (s)
3′	3.02 (ddd, 11.7, 9.0, 5.9)	2.92 (ddd, 12.7, 8.3, 5.4)	3.10 (ddd, 10.1, 9.2, 6.4)	2.79(ddd, 12.5, 8.4, 5.9)
4′α	2.87 (dd, 16.8, 5.9)	2.82 (dd, 16.0, 5.4)	2.70 (dd, 17.0, 6.4)	2.48 (dd, 15.9, 5.9)
4′β	2.62 (dd, 16.8, 11.7)	2.46 (dd, 16.0, 12.7)	2.64 (dd, 17.0, 10.1)	2.43 (dd, 15.9, 12.4)
8′	6.71 (br s)	6.70 (br s)	6.90 (s)	6.88 (s)
10′	6.70 (br s)	6.70 (br s)	6.71 (s)	6.71 (s)
15′	2.42 (s)	2.42 (s)	2.38 (s)	2.38 (s)
16′	3.88 (s)	3.86 (s)	3.74 (s)	3.75 (s)
11-OH	12.04 (s)	12.00 (s)	12.15 (s)	12.12 (s)
2′-OH	4.65 (br s)	4.41 (br s)	7.80 (s)	7.53 (s)
11′-OH	12.34 (s)	12.29 (s)	12.49 (s)	12.47 (s)

[a] Recorded in $CDCl_3$ at 500 MHz.

[b] Recorded in DMSO-d_6 at 600 MHz.

Table S2. ^{13}C NMR spectrum of 4 and epiremisporine B.

Position	4 (2′*S*) [a]	4 (2′*R*) [a]	epiremisporine B (2′*S*) [b]	epiremisporine B (2′*R*) [b]
			δ_C, Type	
1	171.0, C	172.3, C	170.8, C	171.3, C
2	88.7, C	90.7, C	88.2, C	89.5, C
3	48.3, CH	48.2, CH	47.0, CH	46.6, CH
4	37.1, CH	36.4, CH	36.9, CH	36.2, CH
5	168.8, C	168.6, C	169.3, C	168.9, C
7	157.3, C	157.2, C	156.8, C	156.8, C
8	108.3, CH	108.3, CH	108.3, CH	108.3, CH
9	147.3, C	147.5, C	147.5, C	147.5, C
10	113.1, CH	113.2, CH	112.3, CH	112.3, CH
11	160.4, C	160.8, C	159.9, C	159.9, C
12	108.9, C	108.9, C	108.4, C	108.4, C
13	179.1, C	179.0, C	178.9, C	178.9, C
14	119.2, C	118.5, C	119.2, C	118.7, C
15	22.2, CH_3	22.2, CH_3	21.5, CH_3	21.4, CH_3
16	53.2, CH_3	53.6, CH_3	52.8, CH_3	52.6, CH_3
1′	169.4, C	167.3, C	169.4, C	167.7, C
2′	104.8, C	105.9, C	105.7, C	106.2, C
3′	43.4, CH	47.1, CH	42.7, CH	47.3, CH
4′	26.3, CH_2	27.5, CH_2	26.3, CH_2	26.9, CH_2
5′	166.7, C	166.0, C	168.0, C	167.4, C
7′	156.1, C	156.1, C	155.5, C	155.6, C
8′	107.6, CH	107.6, CH	107.6, CH	107.6, CH
9′	147.5, C	147.7, C	147.4, C	147.4, C
10′	112.6, CH	112.6, CH	112.0, CH	112.0, CH
11′	160.4, C	160.8, C	159.5, C	159.6, C
12′	108.4, C	108.5, C	107.7, C	107.8, C
13′	179.8, C	179.7, C	179.4, C	179.3, C
14′	112.6, C	112.4, C	111.9, C	111.7, C
15′	22.4, CH_3	22.4, CH_3	21.8, CH_3	21.8, CH_3
16′	53.3, CH_3	53.1, CH_3	52.3, CH_3	52.3, CH_3

[a] Recorded in $CDCl_3$ at 125 MHz. [b] Recorded in DMSO-d_6 at 150 MHz.

Table S3. The ROESY correlations for compounds 1–3.

Position	1 [a]	2 [a,]	3 [a,]
		ROESY	
3	4, 16, 3′, 4′α	4, 16, 3′, 4′α	4, 16, 3′, 4′α
4	3	3	3
8	15	15	15
10	15	15, 11-OMe	15, 11-OMe
15	8, 10	8, 10	8, 10
16	3	3	3
3′	3, 4′α, 4′β, 2′-OMe	3, 4′α, 4′β, 2′-OMe	3, 4′α, 4′β
4′α	3, 3′, 4′β	3, 3′, 4′β	3, 3′, 4′β
4′β	3′, 4′α	3′, 4′α	3′, 4′α, 2′-OMe
8′	15′	15′	15′
10′	15′	15′	11′-OH, 15′
15′	8′, 10′	8′, 10′	8′, 10′
11-OMe		10	10
2′-OMe	3′	3′	4′β
11′-OH			10′

[a] Recorded in $CDCl_3$ at 500 MHz.
